# Age Induced Nitroso-Redox Imbalance Leads to Subclinical Hypogonadism in Male Mice

**DOI:** 10.3389/fendo.2019.00190

**Published:** 2019-03-28

**Authors:** John Alden Lee, Manish Kuchakulla, Himanshu Arora, Shathiyah Kulandavelu, Evert Gonzalez, Thomas A. Masterson, Joshua M. Hare, Ursula B. Kaiser, Ranjith Ramasamy

**Affiliations:** ^1^Department of Urology, Miller School of Medicine, University of Miami, Miami, FL, United States; ^2^Miller School of Medicine, The Interdisciplinary Stem Cell Institute, University of Miami, Miami, FL, United States; ^3^Division of Endocrinology, Diabetes and Hypertension, Brigham and Woman's Hospital, Harvard Medical School, Boston, MA, United States

**Keywords:** subclinical hypogonadism, compensated hypogonadism, aging, nitroso-redox imbalance, nitrosative stress, 4-hydroxynonenal, S-nitrosoglutathione reductase

## Abstract

**Objective:** The cause of age-related changes in testosterone remains unclear. We hypothesized that increased nitroso-redox imbalance with aging could affect testosterone production.

**Materials and Methods:** We measured several markers of nitroso-redox imbalance (4-HNE, 3-NT, and NT) in serum of S-nitrosoglutathione reductase knock out (GSNOR KO) mice that have increased nitroso-redox imbalance and compared these to wild-type (WT) mice. We evaluated the impact of age-induced nitroso-redox imbalance on serum luteinizing hormone (LH) and testosterone (T) in WT young (<2 months), middle-aged (2–6 months), and aged (>12 months) mice. Finally, to elucidate the susceptibility of testes to nitroso-redox imbalance, we measured 4-HNE protein levels in the testes of WT and KO mice.

**Results:** We identified 4-HNE as a reliable marker of nitroso-redox imbalance, as evidenced by increased protein levels in serum of GSNOR KO mice compared with WT mice. We demonstrated that 4-HNE serum protein levels increase in WT mice with age but do not accumulate in the testes. We also found that T levels were similar in all age groups. Interestingly, we found that serum LH levels in aged and middle-aged mice were increased when compared to young mice (*n* = 5) consistent with the phenotype of subclinical hypogonadism.

**Conclusions:** Increased serum 4-HNE and LH levels without changes in T with age suggest that nitroso-redox imbalance is associated with subclinical hypogonadism in aged mice. Recognizing the relationship and etiology of a currently poorly understood classification of hypogonadism could be a paradigm shift in how age-related testosterone change is diagnosed and treated.

## Introduction

Age-related reproductive and sexual decline is a ubiquitous process. Recently, interest in identifying sources of age-related decline in sexual function in males has grown due to an increasing proportion of older people in the population (1). The hypothalamic-pituitary-gonadal (HPG) axis, which regulates sexual function in males, is responsible for the control of production of luteinizing hormone (LH) and testosterone (T). Serum levels of T change with age (2, 3), and these changes can be due to a combination of testicular (primary hypogonadism, high LH, and low testosterone) and pituitary or hypothalamic (secondary or tertiary hypogonadism, low, or normal LH with low testosterone) failure. Although it has been suggested that hypogonadism effects as much as 23.3% of men aged 40–79 years, the pathophysiology of age-related hypogonadism and its specific classifications have not been well-defined. In one study, in addition to those with low T, an additional 9.5% of men in the study had compensated or subclinical hypogonadism (increased LH and normal T) (4). The mechanism of this type of hypogonadism remains unknown.

Currently, the free radical theory of aging is the most accepted hypothesis to explain factors that contribute to functional decline associated with aging. (5, 6) This theory of aging proposes that reactive oxygen species (ROS), such as superoxide (O2^−^) and hydrogen peroxide (H_2_O_2_) and reactive nitrogen species (RNS) including nitrogen dioxide (NO_2_) and peroxynitrite (ONOO^−^) play key roles by inhibiting cellular function, growth, and apoptosis. (7–13) When ROS and RNS are produced in excess, a situation of oxidative stress, or nitrosative stress ensues, respectively. However, instead of implicating one or the other in biological systems, many have reasoned that the balance between both types of reactive species is responsible for biological effects (14). A disarray in this balance is better known as nitroso-redox imbalance; it has been associated with several degenerative diseases and negative cellular impacts and have further been shown to increase as a function of age (14, 15). We have previously demonstrated that young adult S-nitrosoglutathione reductase (GSNOR) knock-out (KO) mice have decreased circulating T and LH levels compared to wild-type (WT) controls (16). In the absence of GSNOR, these mice develop excess RNS (17), and their lower levels of T and LH suggest that the nitroso-redox imbalance may disrupt hormone production.

What is unknown is whether age-related changes in T are associated with increasing RNS and ROS (nitroso-redox imbalance). We hypothesized that increased nitroso-redox imbalance can occur with aging and be associated with changes in T production. To investigate this relationship, we confirmed that 4-hydroxynonenal (4-HNE), a sensitive marker for oxidative damage (18) can also reliably be used to measure nitroso-redox imbalance. We then evaluated 4-HNE levels in the serum of young and aged WT mice and compared them to young and aged KO mice. We compared the levels of 4-HNE to changes in LH and T levels that could occur with aging in WT mice. Finally, in an effort to identify the mechanism of the effects of nitroso-redox imbalance we measured 4-HNE levels in the testes of WT mice.

## Materials and Methods

### Mice

Male WT mice (C57/BL6) (Jackson Laboratories, Bar Harbor, ME, USA) and mice lacking GSNOR (KO) (The University of Miami Miller School of Medicine, Miami, FL, USA) were used. Pups were genotyped and sacrificed at the indicated ages. Mice were kept in a barrier-protected animal facility with 12-h light-dark cycles. All experiments were carried out before 10 am to reduce the impact of diurnal variation of LH and T levels. Blood was obtained from WT mice by cardiac puncture followed by euthanasia for the young (< 2 months), middle aged (6 month), and aged (12–15 months) time points. We obtained blood via saphenous vein draw for the 2, 3, and 4 month time points. Saphenous vein blood collections were done on the same mice over time as they aged. All saphenous vein and cardiac puncture blood collections were done under isoflurane anesthesia. Euthanasia was performed by cervical dislocation under isoflurane anesthesia. All methods were performed in accordance with the approved institutional animal care and use committee protocol at our institution. All methods were performed with the approval of the University of Miami Institutional Animal Care and Use Committee (IACUC) protocol at our institution (Protocol # 15167).

### Western Blotting

Serum proteins were isolated from total blood and processed with RIPA lysis buffer. Lysates were mixed with 5 × sample buffer, heated to 100°C for 5 min, and separated by 10% odium dodecyl sulfate-polyacrylamide gel electrophoresis (SDS-PAGE). Resolved proteins were transferred onto a 0.45-μm polyvinylidene fluoride (PVDF) membrane. Proteins were studied by exposing the membranes to antibodies against 4-HNE (Alpha Diagnostic International Intl. Inc., HNE11-S), Transferrin (Abcam, ab84036), and Albumin (Abcam, ab19195), 3-Nitrotyrosine (Abcam, ab61392), and Nitrotyrosine (Abcam, ab42789). Immunoreactive bands were visualized using the Thermo Scientific Chemiluminescent Pico Kit.

### Testes Protein Isolation

Testes from mice were dissected, and the tunica albuginea was removed and collected in chilled RIPA buffer (150 mM NaCl. 0.1% SDS, 1% NP-40, 50 mM Tris pH 7.5, 0.5% Sodium-Deoxycholate) for preparation of whole testis protein lysates. Tissues were disrupted mechanically and incubated on ice for 30 min and lysates were cleared from residual cell debris by centrifugation for 10 min at 10,000 × g. The supernatant was collected, and protein concentrations were determined by Bradford colorimetric assay.

### Testosterone and LH Assay

Serum total testosterone and LH levels were measured using the Ligand Assay & Analysis Core of the Center for Research in Reproduction at the University of Virginia (Charlottesville, VA, USA). Techniques for measurements of each hormone are described in detail at https://med.virginia.edu/research-in-reproduction/ligand-assay-analysis-core/assay-methods/. For serum testosterone, the mean intra-assay variation was 12.8% and the intraassay variation was 9.3%. Serum LH was measured using an ultrasensitive enzyme-linked immunosorbent assay. In brief, 6 μL of whole blood was collected in 54 μL of assay buffer for analysis. Intraassay coefficients of variation were 7.3% (low quality control [QC]; 0.13 ng/mL), 5.0% (medium QC; 0.8 ng/mL), and 6.5% (high QC; 2.3 ng/mL). All experiments were performed in the morning to minimize the impact of diurnal variation of hormones.

### Epididymal Sperm Concentration

After the mice were euthanized, epididymides were collected and used for semen analysis. Fresh epididymis was cut into small pieces and dispersed in F12 medium 200 μL (Invitrogen, Waltham, MA, USA) containing 0.1% bovine serum albumin (Invitrogen) pre-warmed to 37°C and incubated for 15 min to facilitate the transmigration of sperm from the epididymis. Each epididymal sperm suspension was subjected to sperm counting and sperm motility analyses by a computer-aided semen analysis (CASA) system (Microptic SL, Barcelona, Spain).

### Statistical Analysis

Data were analyzed for significance using analysis of variance with the Student *t*-test, as indicated. All analyses were performed using GraphPad Prism 4.03 (GraphPad, South San Francisco, CA, USA), and a *P* < 0.05 was considered significant. All data are presented as mean ± standard error.

## Results

### Serum 4-HNE Levels Are a Reliable Marker of Nitroso-Redox Imbalance

**Figure 1:**
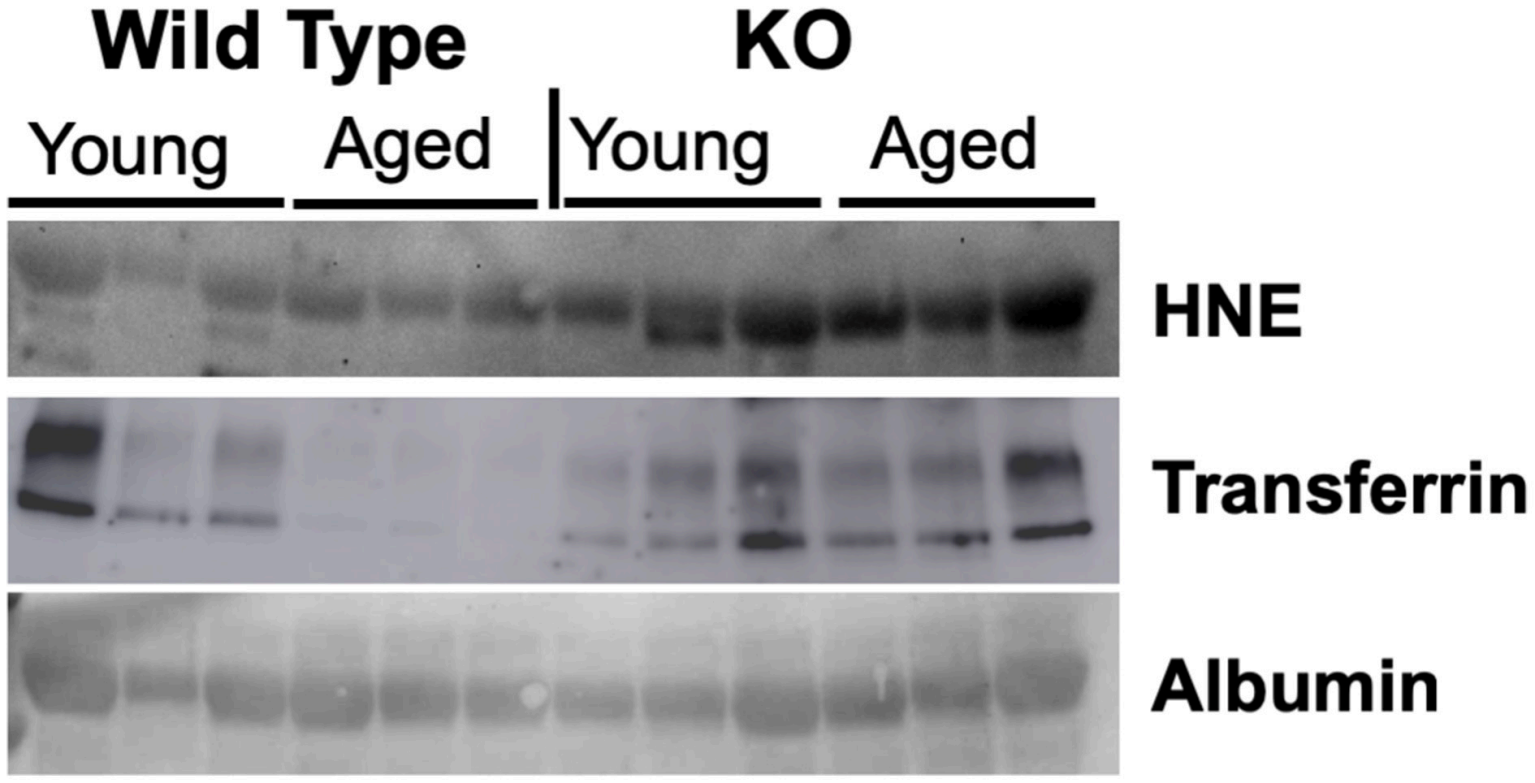
4-HNE is a reliable marker of nitroso-redox imbalance. To identify a reliable marker of nitroso-redox imbalance in serum, we evaluated several markers such as 4-HNE, 3-NT, and NT. Western blot indicated that 4-HNE protein levels are a reliable marker of nitroso-redox imbalance, as evidenced by increased expression in GSNOR KO mice compared to WT mice.

We evaluated markers of nitroso-redox imbalance such as 4-HNE, 3-Nitrotyrosine (3-NT), and Nitrotyrosine (NT) in the serum of WT and GSNOR KO mice. We identified 4-HNE as a reliable marker of nitroso stress as evidenced by higher serum protein levels in GSNOR KO mice compared to WT mice (Figure 1). The other markers, 3-NT and NT, did not demonstrate significant differences in serum protein levels between GSNOR KO and WT mice (data not shown).

### Age Is Associated With Increased Nitroso-Redox Imbalance

**Figure 2:**
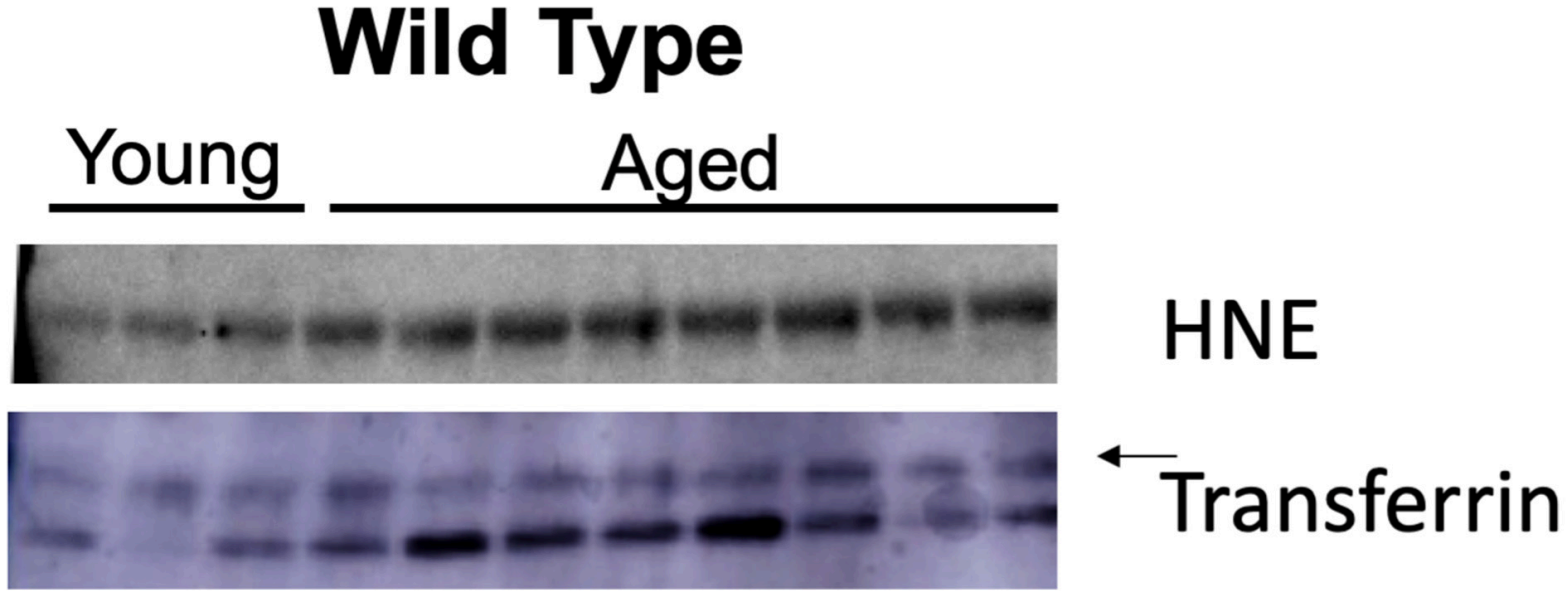
Age is associated with increased nitroso-redox imbalance. To validate that nitroso-redox imbalance is increased with age, we compared 4-HNE serum protein levels in young (<2 months) and aged mice (>12 months). Western blot demonstrated 4-HNE serum protein levels are increased with age.

Nitroso-redox imbalance is associated with increasing age. We investigated whether 4-HNE protein levels increase with age. We evaluated the levels of 4-HNE in serum samples from young (<2 months) and aged (>12 months) mice using Western blot. We found that 4-HNE levels were higher in older mice when compared to young mice (Figure 2). This result suggests that nitroso-redox imbalance increases with age.

### Aging Affects Circulating LH, Testosterone, and Sperm Concentration

**Figure 3:**
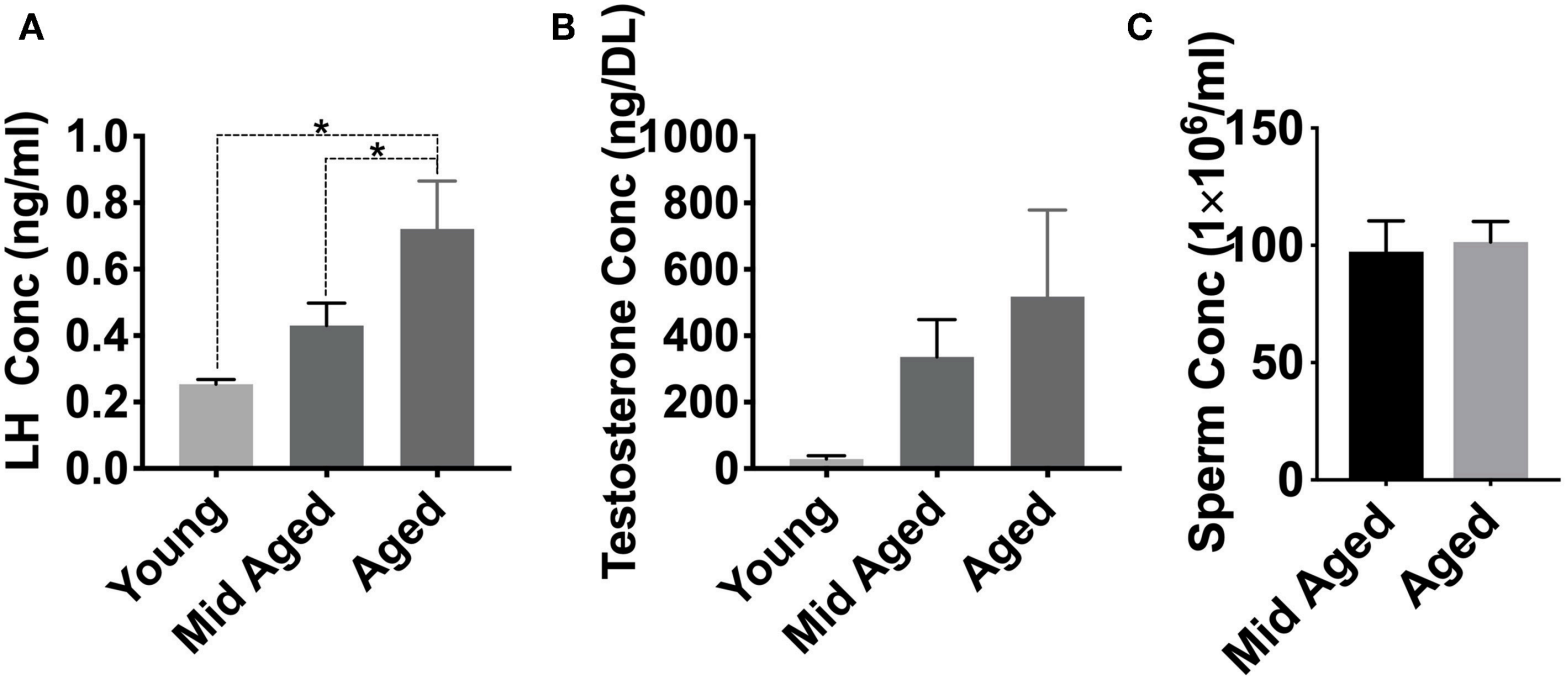
Aging effects on LH and testosterone levels. We examined if age-associated nitroso-redox imbalance impacts serum LH, Testosterone (T), and epididymal sperm concentration. **(A)** LH levels are higher in aged (>12 months) mice than in young (<2 months) or middle-aged (2–6 months) mice (^*^p < 0.05) **(B)** T concentrations show no significant differences with age **(C)** Epididymal sperm concentrations are not different between middle-aged and aged mice.

We have previously demonstrated that nitroso-redox imbalance in GSNOR KO mice decreased serum T and LH levels in addition to impairing sperm parameters, compared to WT controls (16). We examined if age-associated nitroso-redox imbalance impacts LH, T, and epididymal sperm concentration. For these experiments, we measured T, LH, and epididymal sperm concentration in young (<2 months), middle-aged (2–6 months), and aged mice (>12 months) over time. We found that LH levels in aged (*n* = 8) and middle-aged (*n* = 5) were increased when compared to young mice (*n* = 5) (Figure 3A). T levels were not significantly different between any of the age groups (*n* = 5) (Figure 3B). We also did not identify any changes in epididymal sperm concentration (Figure 3C). Collectively, LH concentration increased with age, while T and sperm concentration were not significantly impacted (Supplementary Figure 1). Taken together, these results suggest that age-induced increase in nitroso-redox imbalance is associated with subclinical hypogonadism.

### 4-HNE Protein Levels Are Not Accumulated in the Testes With Aging

**Figure 4:**
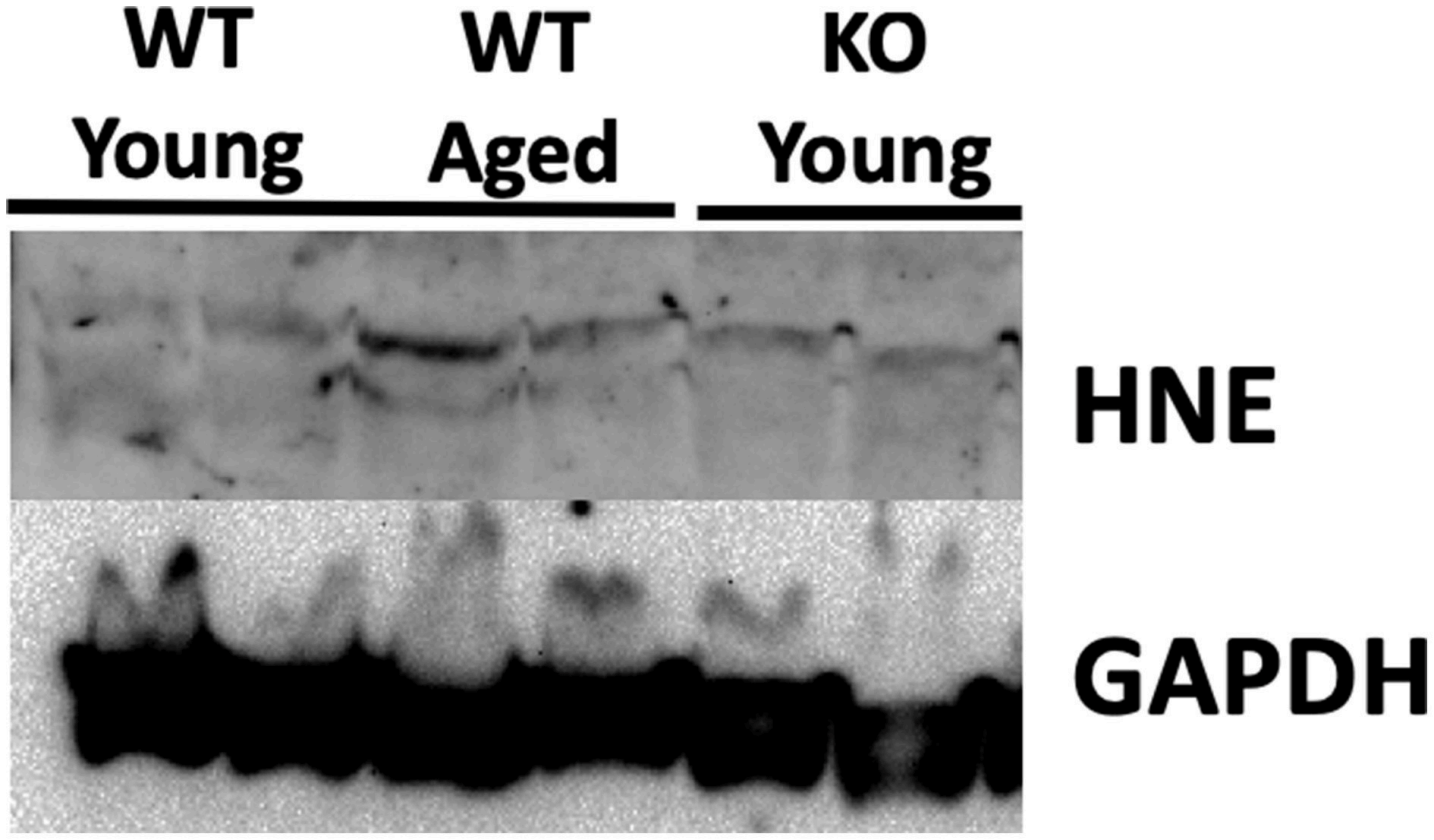
4-HNE protein expression does not accumulate in the testis with age. We measured protein levels of 4-HNE in the testes of young WT, middle-aged WT, and young KO. Protein levels were similar in all of the groups suggesting that 4-HNE protein levels in the testes may not be a reliable indicator of nitroso-redox imbalance.

Previous studies have demonstrated that testicular tissue is particularly susceptible to nitroso-redox imbalance (19). To examine this, we measured 4-HNE protein levels in the testes of young WT, middle-aged WT, and young KO mice. Our results demonstrated that testicular 4-HNE protein levels were similar in all groups (Figure 4). This result suggests that the testis does not accumulate nitroso-redox imbalance with age, or that and that testicular 4-HNE protein levels may not be a reliable indicator of nitroso-redox imbalance.

## Discussion

The mechanism of age-associated changes in testosterone production remains unclear. Accumulating free radicals with nitrosative and oxidative stress can lead to cellular aging. We investigated whether increasing nitroso-redox imbalance with aging can explain changes in T. We identified that age is not only associated with increasing nitroso-redox imbalance as reflected by serum 4-HNE protein levels but is also associated with increased LH with unchanged T levels, suggesting a profile of compensated hypogonadism. As this imbalance increases with age, T production can decrease, resulting in a reduction of the negative feedback inhibiting LH production. The result is an increase in LH that is enough to stimulate steroidogenesis to produce T levels in the normal range; increased LH “compensates” for the decreased T production. As a result, T levels remain within normal reference ranges concurrently with elevated LH levels. While the mechanism remains uncertain, our results indicate that Leydig cells capacity for T production is not diminished since T levels remain in normal range. However, the mechanism may lie in the HPG axis in the Leydig cells ability to respond to LH and stimulate T production. Thus, we suggest that nitroso-redox imbalance may lead to compensated hypogonadism through effects of the HPG axis. Our results suggest an association between nitroso-redox imbalance and subclinical hypogonadism, but the cause must still be elucidated.

Compensated hypogonadism, also referred to as subclinical hypogonadism, has been suggested to be its own clinical condition (20, 21). The overall clinical and physiological significance of compensated hypogonadism is poorly understood, but it has been found to be associated with both aging and increased physical symptoms of T deficiency (22). This association, combined with its surprising prevalence in men [9.5% of all men aged 40–79 (4)], raises the question of whether this classification of hypogonadism should be diagnosed or potentially even treated. Men, and especially older men, may benefit from having LH included in the initial screening for hypogonadism in order to diagnose subclinical hypogonadism. It also should be investigated whether treating subclinical hypogonadism, much like treating subclinical hypothyroidism, can be of benefit. In this study, we show that increased levels of nitroso-redox imbalance occur with age suggesting a possible etiology for compensated hypogonadism in older men. This discovery provides an aim for future studies to evaluate if measuring nitroso-redox imbalance in older men may be of diagnostic benefit by identifying a potential therapeutic target in the aging process. Novel therapeutic strategies such as anti-oxidant therapy, may have a significant impact on treating subclinical hypogonadism. Since subclinical hypogonadism represents a form of testicular failure future studies should continue to investigate the mechanism of nitroso-redox imbalance at the level of Leydig cell steroidogenesis and whether treating the nitroso-redox imbalance could reverse these HPG axis changes and potentially prevent a progression of testicular failure resulting in primary hypogonadism.

Our study has strengths and limitations. A strength of this study is that it is the first study to show that 4-HNE can be a reliable marker for nitroso-redox imbalance. This study also demonstrates that nitroso-redox imbalance increases with age. Limitations of the study include the small sample size (limited by the breeding capabilities of GSNOR KO mice) and variability in serum LH and T levels in mice. Mice are also known to have variable T levels due to lack of circulating sex hormone binding globulin (23). We accounted for this variability by consistently drawing the blood in the morning (before 10 a.m.).

## Conclusion

Aging is associated with increased nitroso-redox imbalance as reflected by serum 4-HNE protein levels and subclinical hypogonadism. Recognizing the relationship and etiology of a currently poorly understood classification of hypogonadism could be a paradigm shift in how we diagnose and treat age-related hypogonadism. Future studies can focus on identifying markers of nitroso-redox imbalance in testis and the mechanisms of how they impact steroidogenesis.

## Data Availability

The datasets generated for this study are available on request to the corresponding author.

## Author Contributions

JL, MK, HA, SK, TM, JH, UK, and RR designed the research. JL, MK, HA, and EG performed the research. JL, MK, HA, SK, and RR analyzed the data. JL, MK, and RR wrote the paper.

### Conflict of Interest Statement

JH discloses a relationship with Vestion, Inc., that includes equity, board membership, and consulting; is the chief scientific officer, a compensated consultant and advisory board member for Longeveron, and holds equity in Longeveron; and is the co-inventor of intellectual property licensed to Longeveron. The remaining authors declare that the research was conducted in the absence of any commercial or financial relationships that could be construed as a potential conflict of interest.

## Supplementary Material

Figure S1 LH and testosterone concentrations further broken down by age show the linear increase in LH concentration as the mice aged.

## Abbreviations

WT: wild-type
GSNOR: S-nitrosoglutathione reductase
LH: luteinizing hormone
HPG: hypothalamic-pituitary-gonadal
ROS: reactive oxygen species
RNS: reactive nitrogen species
KO: knock-out
4-HNE: 4-hydroxynonenal
3-NT: 3-Nitrotyrosine
NT: Nitrotyrosine.
