# Supplementary figures and images for: Age Induced Nitroso-Redox Imbalance Leads to Subclinical Hypogonadism in Male Mice
Source: Front Endocrinol (Lausanne). 2019 Mar 28;10:190. doi: 10.3389/fendo.2019.00190 (PMC6447610; doi:10.3389/fendo.2019.00190)

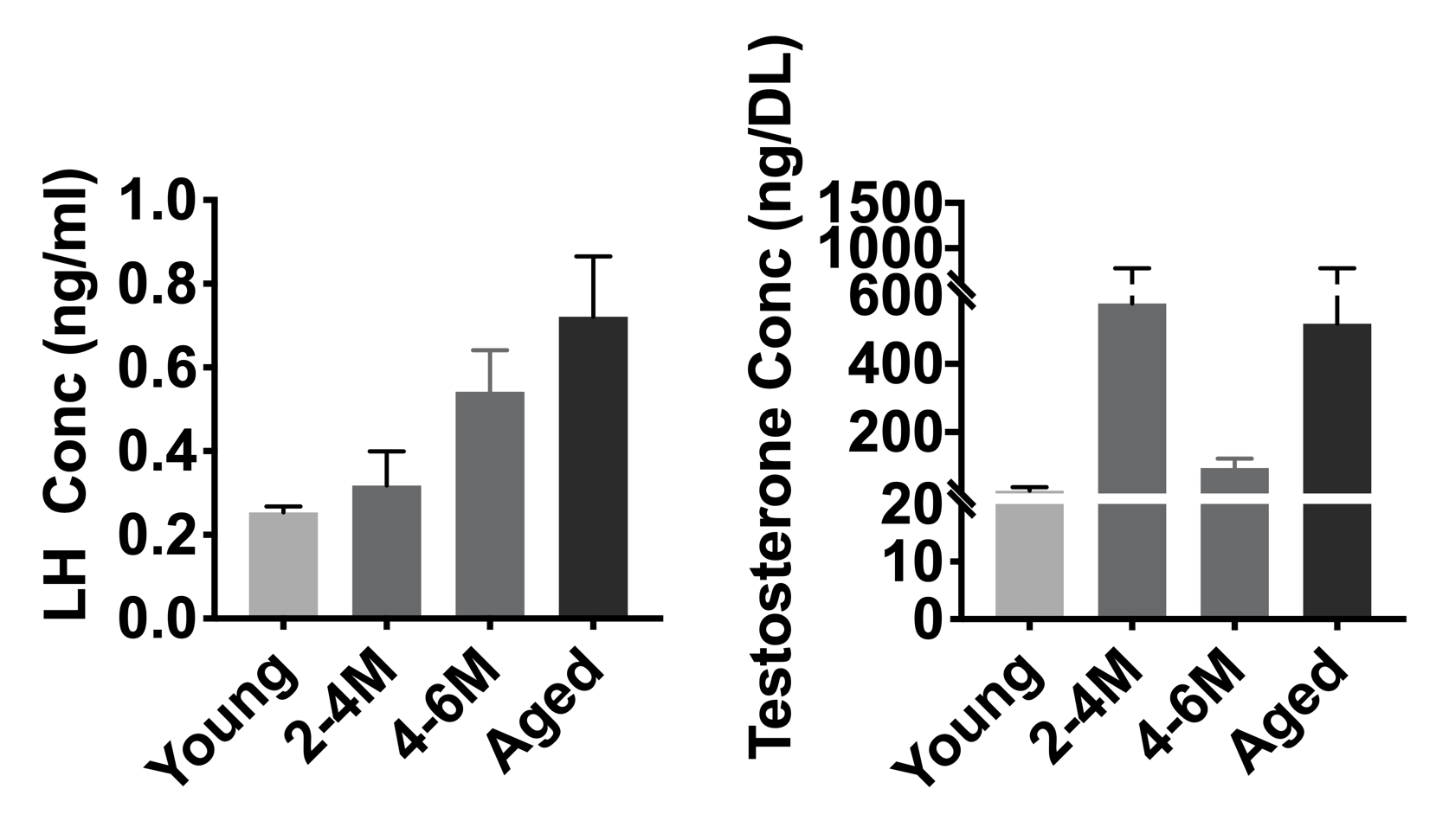

Supplement: Figure S1 — LH and testosterone concentrations further broken down by age show the linear increase in LH concentration as the mice aged. [file Image_1.TIFF]
